# Examining the ‘Consumer Repair Journey’ and Business Intervention Opportunities to Enhance Customer Experience and Satisfaction

**DOI:** 10.1007/s43615-026-00773-x

**Published:** 2026-04-13

**Authors:** Tung Dao, Tim Cooper, Matthew Watkins

**Affiliations:** 1 De Montfort University, Leicester, UK; 2 Nottingham Trent University, Nottingham, UK; 3 Loughborough University, Loughborough, UK

**Keywords:** Consumer repair journey, Repair behaviour, Business intervention, Product lifetime, Sustainable consumption, Sustainable business

## Abstract

The current linear economic model has led to the creation of low-priced and short-lived products at the cost of shaping fast and unsustainable consumption. Product repair, the process of maintaining the functionality of items by correcting a defect, should, in theory, retain product value and improve material efficiency. However, the transformation of current repair practices requires the comprehensive engagement of consumers and different business stakeholders, including manufacturers, retailers and repair service providers. In particular, the repair journey that consumers go through when deciding whether (and how) to repair an item involves a set of complex decisions and actions. This paper aims to provide a synthesis of this sequence and address the complexity of the consumer’s repair journey, considering different product sectors. To achieve this aim, the research consisted of a set of consumer focus groups to gather insights into consumers’ repair journeys, considering four stages: identification of product faults and repair need, information search and evaluation of alternatives, repair in action and post-repair evaluation. The paper contributes to the field by developing a holistic ‘consumer repair journey’ framework and presents findings on the factors that influence consumer intentions, how these intentions translate into behaviour at each stage, and overall satisfaction during the journey. Additionally, it provides guidance to industry professionals on how to implement interventions that could positively impact consumer repair intentions and their translation into behaviour. These interventions could align with companies’ strategic efforts to embrace customer experience, ‘right to repair’ initiatives, and the growth of sustainable, repair-focused enterprises across various product industries, as advocated by the European Commission (2020) and, more recently, Right to Repair Europe (2023).

## Introduction

A wide range of factors influence consumers’ repair decisions. For example, barriers to repair work characterised by the product, its owner, and the context [11] can generate ‘a gap between the willingness to engage and actual engagement’ [21] or differences between consumers’ decisions and their actions [5, 39, 57]. However, there is a literature gap concerning the steps that most consumers go through before deciding whether to repair faulty products. No previous studies have considered the entire journey that consumers go through before, during and after deciding whether to repair items (including whether to choose a DIY or commercial route).

**Fig. 1:**
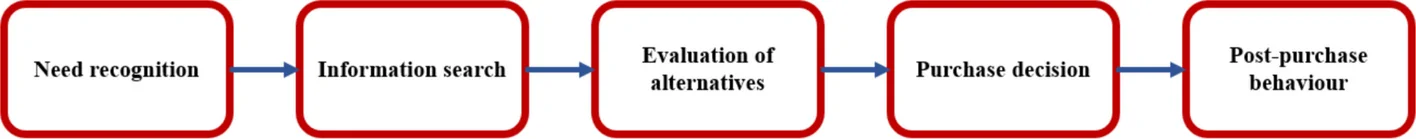
Buyer decision process [16]

Repairing products effectively demands a paradigm shift in how businesses across the value chain sell and interact with products, while also encouraging consumers to choose repair in order to preserve product value. In studying consumers’ repair journeys this research demonstrates novel use of the Five-Stage Model, that Dewey [16] developed to explore the buying decision process from a consumer perspective (Fig. 1). In other words, this study applies this model to the decision-making process for repair that consumers go through when they have a faulty product, developing the concept of a 'consumer repair journey'. Using this concept, the recent study aims to investigate consumers' intentions and the factors influencing the translation of these intentions into behaviour throughout their repair journeys. The study contributes to understanding the importance of business support, such as product design, the provision of repair information, spare parts, and the quality of after-sales services, in shaping consumer repair intentions and behaviour. It also highlights how these factors influence different stages of the repair process across diverse consumer demographics, including various genders and generations.

Currently, eco-design repairability standards are being formulated for a limited range of products such as washing machines, dryers, dishwashers, fridges, displays, welding equipment and vacuum cleaners. Right to Repair Europe [63], is a coalition representing over 170 organisations from 27 European countries, who have lobbied the European Commission, urging them to expand standards for a broader range of products and called for the inclusion of more Information and Communications Technology and other electronic products, which are often considered the most problematic and challenging to repair. This study aims to furnish evidence relating to their appeal ‘*Call to the EU: we need to extend the lifetime of all electronics’* [63], it also covers a broader spectrum of product sectors, notably furniture and clothing.

## An Overview of Past Literature on Repair from a Consumer Perspective

### Consumer Buying Process and Repair Journey

The Five-Stage Model of Dewey [16] is used to evaluate customers' buying decision process (Fig. 1). An actual purchase decision is part of the buying process and capturing and understanding the process can help brands or marketers to intervene and influence their customers’ purchases [43]. The current research demonstrates novel use of this research approach to explore (i) the repair journey that most consumers go through and (ii) opportunities for business interventions based on consumer intention and behaviour at each stage of the journey.

A consumer’s buying process often starts with the need recognition stage, which normally identifies what the problem or need is, whether the product or service is required, and how the consumer is likely to feel after making the purchase [41, 43]. The information search stage commences when consumers put effort into identifying and observing various sources of information, to support their buying decisions. However, some consumers may not seek additional information if their motives for purchase are significant and satisfying products are near at hand [43].

At the stage of evaluating alternatives, consumers often compare different products or brands’ attributes, considering whether they can deliver acceptable value [42]. To enhance the probability of being considered at this juncture, brands should comprehend the benefits preferred or sought by consumers, as well as which product attributes will exert the most significant influence on the consumers’ decision-making process. The fourth stage is when the purchase takes place. During this time, consumers may form an intention to buy the most preferred product or brand because they have evaluated all the alternatives and estimated the value of the items or services to them.

The final stage is when customers assess whether they are satisfied or dissatisfied with their purchases. This post-purchase behaviour may influence future purchases [16] – whether to buy the product again or alternatives (either similar products from other brands or substitutes). Businesses may want to engage their customers with post-purchase communications, such as through customer satisfaction surveys or targeted marketing (e.g. sending birthday greetings), to influence consumers’ feelings about their past and future purchases.

Later studies have recommended enhancing the buyer decision process to encourage purchases and improve consumer experiences [1, 71]. However, the promoted approach (i.e. easing purchase process) might also lead to unsustainable consumption, prompting more frequent purchases and a preference for replacement over repair. The Health and Safety Executive (HSE) and WRAP have addressed concerns about the waste generated from the production and consumption of items such as electrical and electronic products, furniture and textiles and consequent harm to the environment, estimating that 2 million tonnes of electrical and electronic equipment [33], 670,000 tonnes of furniture [74] and 921,000 tonnes of clothes being disposed of in the UK annually, on average [77]. The environmental benefits of repair can be achieved by delaying items from being discarded; evidence suggests that many discarded items could be reused directly or after minor repair [74].

**Fig. 2:**
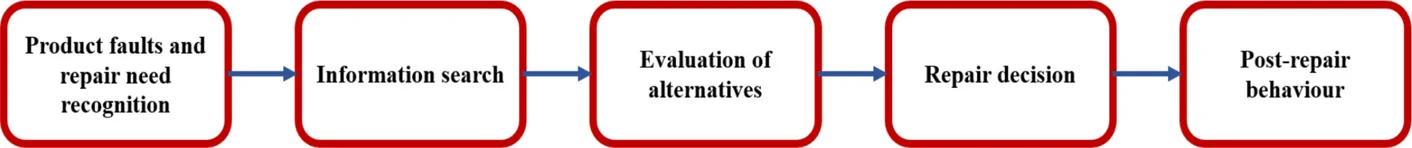
The proposed consumer repair journey (before the pilot study)

The current study presents a conceptual consumer repair journey (Fig. 2) inspired by the Five-Stage Model [16]. It departs from a focus on understanding the purchasing process, instead exploring the factors that influence repair,specifically, the consumer’s intention, behaviour and experience throughout the repair journey. The definitions of the five stages were developed based on insights from two primary references which, together, significantly contribute to shaping the proposed consumer repair journey. Initially, the five stages (Fig. 2) emerged from discussions with researchers in the sustainable consumption field from institutions that included Nottingham Trent University, Japan’s National Institute for Environmental Studies, Berlin Institute of Technology (Technische Universität Berlin) and Aalborg University, alongside repairers affiliated with The Restart Project (therestartproject.org) and Nottingham Fixers (www.facebook.com/NottinghamFixers). Both The Restart Project and Nottingham Fixers organise community repair events aimed at reducing waste by extending the lifespan of consumer products. Then, feedback was gathered from a focus group comprising consumers who participated in a pilot study for this project, and who also possessed research expertise in sustainable consumption.

**Fig. 3:**
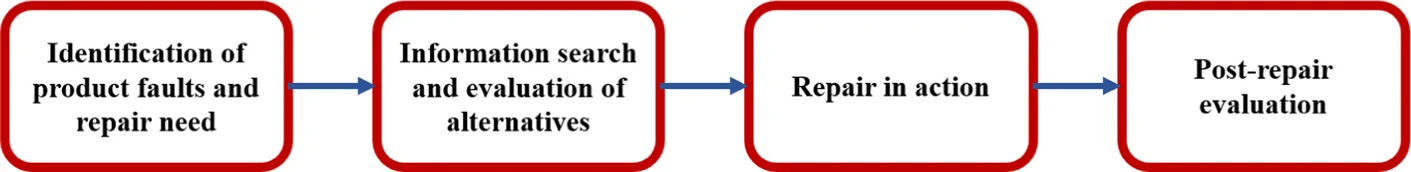
The modified consumer repair journey (after the pilot study)

The pilot study group discussion resulted in a consensus on the consumer repair journey, condensing it into four stages (Fig. 3) instead of five.

At the first stage of the consumer repair journey, owners often identify potential issues with their products, determining if they require repair due to personal stimuli (e.g. prior knowledge about repair or emotional attachment to specific items) or situational factors such as the availability of troubleshooting manuals. Marketers commonly use advertisements to stimulate consumer desires, either encouraging new product purchases or promoting DIY or professional repair services [42].

At the second stage, consumers seek information on DIY repair methods or repair service options. They may utilise online reviews. However, some prioritise specific resources (e.g. YouTube tutorials, repair blogs or manufacturer websites) based on perceived reliability shaped by their past experiences [7]. Thus, this study aims to identify sought-after repair information and preferred sources. Moreover, consumers contemplate alternatives at this second stage, including replacement, utilising various assessment methods. While evaluations can be rational, involving comprehensive examinations of online reviews and seeking advice, they may also be influenced by situational factors such as price and store ambience [43]. Adverse feedback from other customers could also impact decisions.

At the third stage, consumers decide whether to self-repair, seek professional repair services, or opt for replacement, influenced by situational factors such as price and online reviews. This paper explores how these factors affect consumer choices and actions regarding repair. Finally, at the fourth stage, consumers may evaluate the outcomes of their repair decisions, determining satisfaction levels.

Previous study suggested that the buying decision process is rarely linear; consumers often revisit earlier stages based on their experiences and shifting needs [43]. Therefore, this research investigates trends such as stage continuity or repetition during consumer repair journeys.

Many studies have illuminated opportunities for and challenges to the repair of electrical and electronic equipment (EEE) [5, 8, 10, 18, 20, 25–27, 31, 47, 58, 60–62, 66–69, 73] and clothing [3, 4, 14, 19, 24, 28–30, 35, 36, 48, 49, 51, 75, 76], although furniture studies have been more limited (e.g. [34],European Environmental Bureau (EEB), [23]). However, there is a dearth of literature which would map consumer repair journeys across different product sectors [64]. Although recent quantitative research [17] has examined repair decisions across various sectors, it has not investigated the complexity of intention and behaviour at each stage of the consumer repair journey. Given this lack of knowledge, and the substantial volume of waste from discarded EEE, clothing, and furniture, the current research has concentrated on these product categories to better understand the consumer repair journey and its implications for sustainable business interventions.

## Research Method

Constructivists emphasise the role of human interaction in shaping the meaning of a situation and qualitative approaches to research are well-suited to support this process [13]. In the context of the current study, a constructivist worldview was applied to consumer focus groups to explore how participants interpreted their experiences within the consumer repair journey. This approach facilitated discussion amongst participants, encouraging them to build on each other’s ideas [45] and to express the challenges and opportunities within the consumer repair journey in their own words. Creswell and Poth [12] suggests that a focus group process replicates more accurately than interviews how opinions and ideas (i.e. regarding repair journeys) are formulated and exchanged. Participants were able to work together to identify and map out key decisions and actions at each stage of their repair journeys. They shared personal experiences, debated the difficulties encountered, and highlighted opportunities to improve the repair journey, both individually and collectively. Consequently, the constructivist worldview was applied to interpret the data and to deliver key insights for the intended audiences: policymakers, business professionals, environmental advocates and the general public. Bryman [9] recommends that the number of participants in a focus group should range between six and ten. Thus, for each group session researchers aimed to recruit, on average, eight participants. This sample size was considered optimal for facilitating meaningful interaction and ensuring that every participant had the opportunity to contribute to the discussion.

This study focused on Generation X consumers, born between 1965 and 1981, and Generation Y consumers (or ‘Millennials’), born between 1982 and 1998, to investigate differences and similarities in their repair journeys. The rationale for choosing these two generational cohorts were their substantial consumption power, making them critical stakeholders in understanding the dynamics of product repair. Recent reports from World Data Lab [55] found that Generation X had the highest spending of any generation and would maintain its lead through at least 2033. A report by Mintel [53] recognises Generation Y as a major market segment, trending towards value-centric spending, sustainability and tech-oriented consumption. Table 1 outlines the number of participants in each focus group along with their demographics; Group 4’s participants were selected to balance the representation of both generations.

**Table 1:** Focus groups and their demographics

Group	Size	Generation		Sex	
		X	Y	Male	Female
1	10	1	9	2	8
2	7	1	6	2	5
3	8	4	4	5	3
4	9	9	0	5	4
Total	**34**	**15**	**19**	**14**	**20**

Paper- and e-posters for participant recruitment (Appendix 1) were circulated in Nottingham, in targeted Facebook groups (e.g. residential community groups), and in high street shops and restaurants. Participants were recruited via the convenience sampling method [70] and asked to complete a registration form with screening questions (Appendix 2), ensuring that recruitment was efficient and accessible. To further streamline the process, interested individuals were required to complete a registration form, which included screening questions designed to confirm their eligibility and align them with the appropriate focus group based on their demographic information (Appendix 2). This careful approach to recruitment ensured that, overall, the focus groups were representative and conducive to rich, generationally relevant discussion on consumer repair journeys.

The four consumer focus groups were designed to last less than 120 min each and were carried out in Nottingham – a medium sized university city in the UK. Four groups were deemed optimal as a saturation point was identified in the fourth focus group session, at which point no new information or themes emerged. The original five-stage model was presented to participants, and the majority agreed on the merger of stages 2 and 3. Specifically, many participants in the group discussions sought information about different repair routes (Table 3) and their alternatives. They evaluated these options simultaneously as they needed information to support their decisions. Participants considered various alternatives, including leaving the item unrepaired or choosing a replacement with new, refurbished, or pre-owned products. As a result, Stage 3 was renamed 'Repair in action' (instead of 'Repair in decisions' in the five-stage model) to better elucidate the complexity of decisions and actions at this stage. Despite making decisions, participants still experienced intention-behaviour gaps, indicating that even after forming intentions, the transition to taking action remained challenging.

All the focus groups were audio recorded, transcribed and analysed, making use of thematic analysis and NVivo software version 11. The research followed the six phases of thematic coding analysis as outlined by Braun and Clarke [6]. Phase One involved familiarising the investigator with the data by transcribing interview recordings, repeatedly reading the transcripts, and taking notes simultaneously. In Phase Two, initial codes were generated by identifying and coding interesting features across the entire dataset. During this phase, the investigator also employed a deductive coding approach, using a 'start list' of codes and sub-codes [52]. The list of codes (shown in the first column of Table 2) was derived from the stages of the consumer repair journey. Although Dewey's original model outlines five stages, this analysis identifies four stages and six codes, with two of those codes further subdivided into subcategories. The list of sub-codes (the second column of Table 2) were developed from three research questions – (i) what they intended or planned to do, (ii) what they finally decided to do, and (iii) what, if anything, made them change their minds and key variables (e.g. initial intention, behaviour and factors influencing intention or the translation of intention into behaviour).

**Table 2:** Codes and sub-codes

Codes	Sub-codes
Stage 1/ Fault identification	SC1.1: Initial intention (i.e. planned to do)
	SC1.2: Behaviour (i.e. decided to do)
	SC1.3: What influenced intention
	SC1.4: What influenced the translation of intention into behaviour
Stage 1/ Repair needs	SC1.5: Initial intention (i.e. planned to do)
	SC1.6: Behaviour (i.e. decided to do)
	SC1.7: What influenced intention
	SC1.8: What influenced the translation of intention into behaviour
Stage 2/ Information search	SC2.1: Initial intention (i.e. planned to do)
	SC2.2: Behaviour (i.e. decided to do)
	SC2.3: What influenced intention
	SC2.4: What influenced the translation of intention into behaviour
Stage 2/ Alternative evaluation	SC2.5: Initial intention (i.e. planned to do)
	SC2.6: Behaviour (i.e. decided to do)
	SC2.7: What influenced intention
	SC2.8: What influenced the translation of intention into behaviour
Stage 3/ Repair in action	SC3.1: Initial intention (i.e. planned to do)
	SC3.2: Behaviour (i.e. decided to do)
	SC3.3: What influenced intention
	SC3.4: What influenced the translation of intention into behaviour
Stage 4/ Post-repair evaluation	SC4.1: Initial intention (i.e. planned to do)
	SC4.2: Behaviour (i.e. decided to do)
	SC4.3: What made consumers satisfied with the translation of intention into behaviour
	SC4.4: What made consumers dissatisfied with the translation of intention into behaviour

**Fig. 4:**
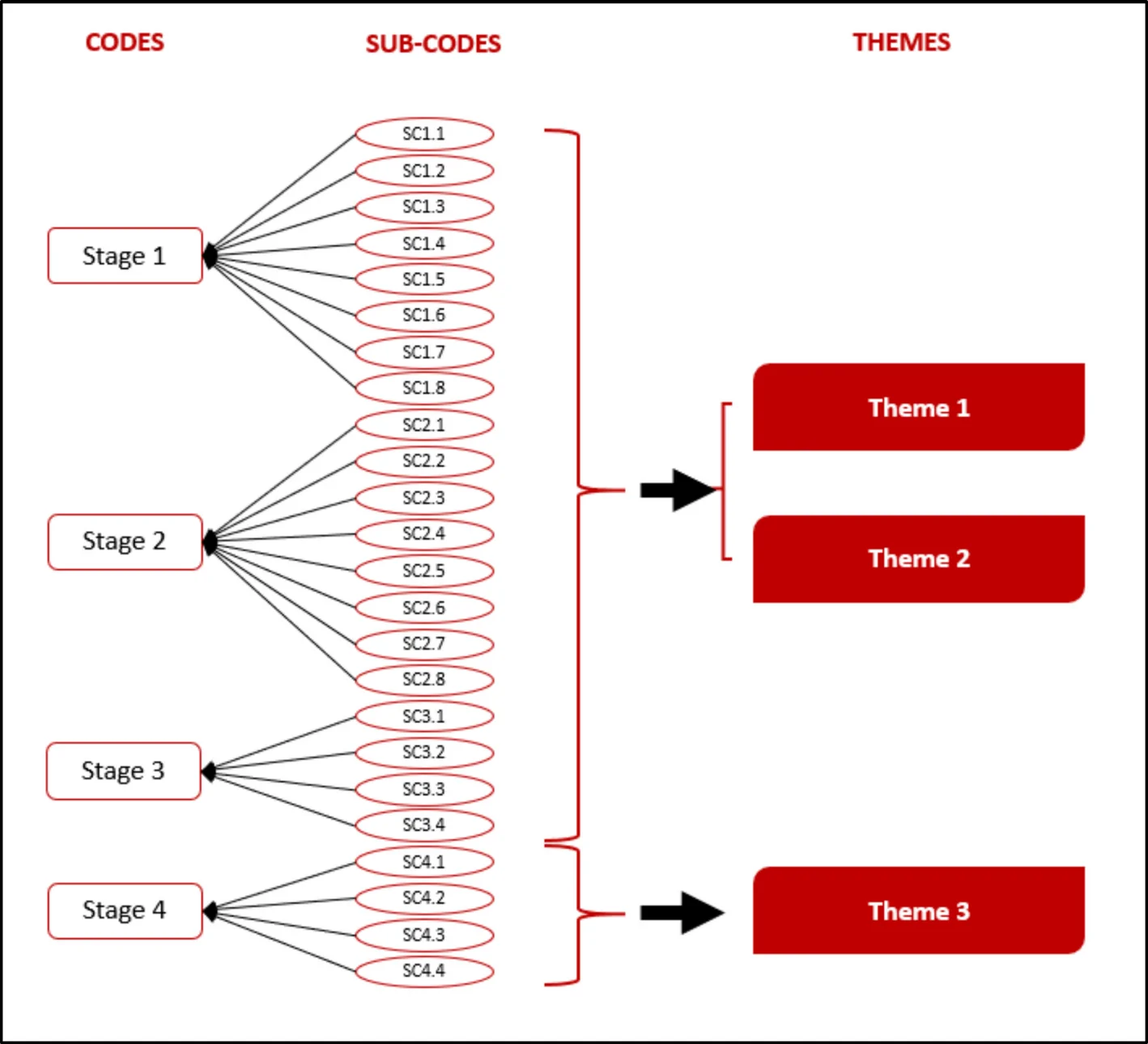
Themes emerging from the Codes and Sub-codes

In Phase Three of the thematic coding process, the focus was on searching for themes by grouping relevant sub-codes into potential themes. Phase Four involved reviewing these themes and constructing thematic networks. Figure 4 presents how three themes emerged from the analysis.

In Phase Five, the themes were clearly defined, named and refined based on ongoing analysis, ensuring their consistency and appropriateness across group discussions. As part of this analysis, any ambiguities or inconsistencies were addressed by revisiting the raw data, conducting additional discussions among the research team, and refining the themes to better reflect the complexity of participants’ experiences. The three themes that emerged were (i) factors influencing the initial intention during the consumer repair journey, (ii) factors influencing the translation of intention into behaviour during the consumer repair journey and (iii) factors affecting the level of satisfaction with the translation of intention into behaviour during the consumer repair journey. The first two themes concentrate on the factors that influence consumer intention and behaviour (both prior to and during the repair actions), while the third focuses on elements impacting the outcomes of those actions.

In Phase Six, the themes and findings from the analysis were integrated and interpreted to generate the insights presented in this paper. This phase involved synthesizing the core themes into a comprehensive narrative that not only answered the research questions but offered practical implications for policymakers, business professional, environmental advocates and the general public.

## Findings: Consumer Repair Journey

This section presents results of group discussions that explored impact factors on consumers’ intention and the translating of intention into behaviour throughout their repair journeys. Despite making decisions, participants still faced intention-behaviour gaps, suggesting that transitioning from intention to action remained difficult. This refinement better captured the complex and evolving nature of consumer repair behaviour. 

Table 3 shows key repair routes and repair identified during the discussions.

**Table 3:** Repair routes and agents

Repair route	Repair agent
Self-repair	DIY product owner
Commercial repair	Retailer
	Independent repairer at a local shop
	OEM agent
Non-commercial repair	DIY family member or friend of product owner
	DIY repairer at a community event or facilitated repair event

### Stage 1 – Identification of Product Faults and Repair Need

**Fig. 5:**
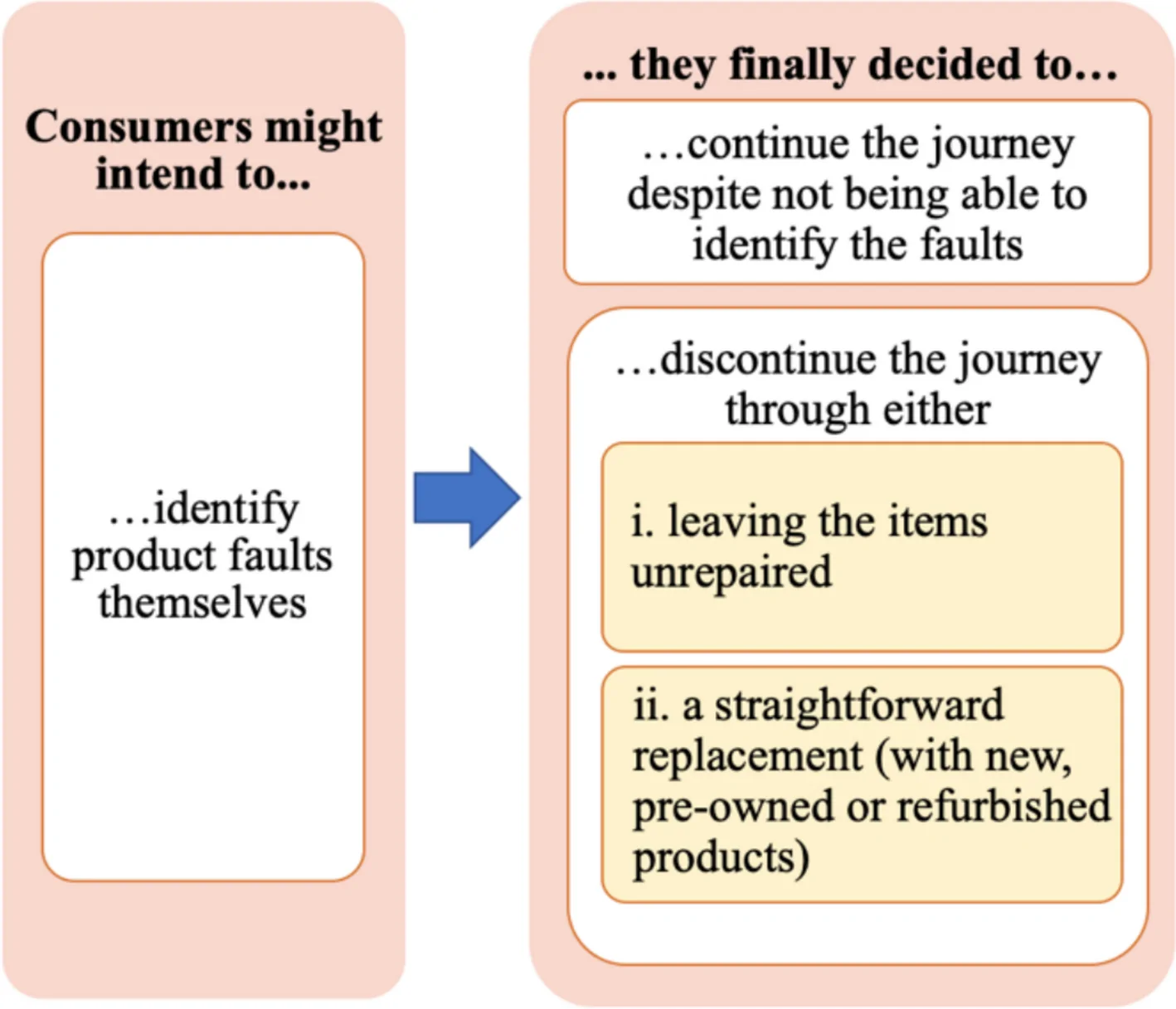
Intention-behaviour gaps at Stage 1

During this stage consumers identify product faults and evaluate whether a repair is necessary. Figure 5 illustrates potential intention-behaviour gaps in the process of identifying product faults. The left-hand box shows intention, coded as SC1.1 in Table 2, whilst the right-hand box presents consumer behaviour (SC1.2), contrasting the absence of a gap (hence continuing the journey), at the top, with reasons as to why a gap might occur (hence discontinuing the journey), at the bottom.

Group discussions suggested that easily recognised product faults, consumers’ interests in repair, high levels of competence and confidence about successful outcomes were key impact factors which determined consumers’ intention to identify product faults themselves, and translating this intention into actual repair behaviour (coded as SC1.3 and SC1.4, respectively). Most focus group participants initially discovered the faults of broken items themselves, especially with garments and furniture. These faults included surface defects, missing or damaged components, and tears or loose stitching, which were more conspicuous in these product categories compared to electrical and electronic equipment (EEE). In the case of EEE, common faults included short circuits, overheating, loose connections, and power supply issues.

The discussions suggested that males tend to be more interested in EEE repair, whilst females are keener on mending clothes – *‘You’ve got something you enjoy more than others [other items].’.* For example, more males indicated that they had identified the faults of EEE, whereas more females succeeded in identifying faults with clothing items. There seemed to be no clear difference between sexes when discussing fault identification of furniture repair.

Participants faced common obstacles in identifying product faults, including a lack of skills, knowledge, or confidence (SC1.4). These challenges led to complex decisions. For instance, some female participants, despite intending to diagnose faults themselves, eventually sought help from friends or commercial repairers due to the identified challenges. This means they continued their repair journeys even though they had not been able to identify the fault or, as expressed by one participant, *‘did not even know where to begin’*. They sought information and help from other actors (e.g. family members, friends, or colleagues) to make a more informed decision at Stage 2.

Some participants were afraid that diagnostic work might lead to further functional, or aesthetic, damage (SC1.4). Particularly, females were more apprehensive about causing functional damage to EEE whilst males were more concerned about further aesthetic damage to textiles. The data under SC1.4 also suggested that fear of data loss was a considerable concern for those who intended to self-identify problems affecting digital devices, particularly when disassembly was necessary. Moreover, a requirement for tools could challenge identification of faults. For example, to find and buy an appropriate screwdriver to use with screws of an uncommon size or shape, such as those with a triangle-shaped head, might require an unacceptable time or financial commitment.

Repair needs (i.e., the type of work required) – coded as SC1.5 – were mainly characterised by owners’ perceptions of the monetary value of products and concerns about time constraints (SC1.7). Consequently, several participants gave up on the journey, leaving their items unrepaired or purchasing a replacement (SC1.6), either before or after trying to identify product faults, due to the perceived challenges e.g. repair costs appeared expensive and uneconomical, or repair work seemed time-consuming (SC1.8). Intentionally buying inexpensive items seemed to lead to simple replacement (SC1.7) when faults with the product were detected, particularly for small appliances (e.g. kettles and toasters), flat-packed furniture and on-trend or fast fashion products. This practice reflects a facet of the current linear economic model, which has generated low-priced and fashionable goods but has also fostered rapid and unsustainable consumption habits.

### Stage 2 – Information Search and Evaluation of Slternatives

During this stage consumers seek information to assist their decisions on whether to repair broken items and, if so, how. The discussion indicated that, after the information search and evaluations of different repair routes, consumers could either keep their intended repair route or change their intention (i.e. choose another repair route or terminate their repair journey). However, there was evidence that some consumers might skip this second stage if they already had knowledge or experience of repairing a product with the same or similar problem.

**Fig. 6:**
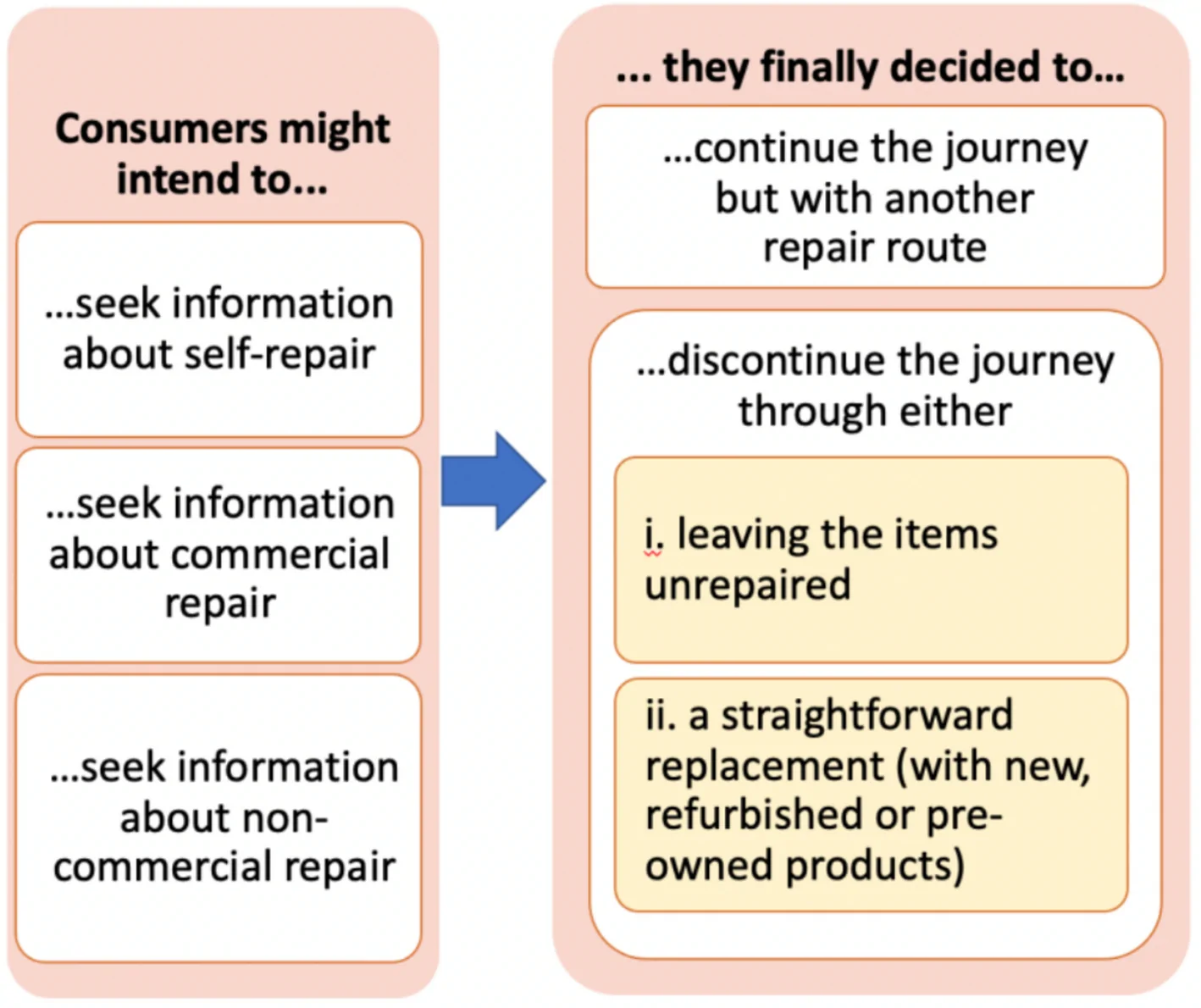
Intention-behaviour gaps at Stage 2

Figure 6 captures the intention-behaviour gaps at this stage. Intention (SC2.1, SC2.5 in Table 2) is shown on the left; consumer behaviour (SC2.2, SC2.6) is on the right, with reasons for the gaps highlighted.

Several participants who intended to do DIY repair often read manufacturers’ manuals, although some females complained about the usefulness of the information provided. A few female participants who lacked repair experience indicated that they relied on their friends’ or family members’ competences and previous experience (SC2.3 and SC2.4).

Several female participants sought instruction or advice from repairers at local shops or customer service staff at the manufacturer or retailer. In contrast, more males preferred to be independent, expressing greater confidence in their existing knowledge and relying on self-directed learning through online sources. The discussions also suggested that it was challenging to find reliable repair service providers. Although some relied on reviews such as those identified through Google Maps, others doubted the reliability of reviewers.

At this stage, most participants expected to get more information to support their decisions on whether and how to repair broken items; however, some left items unrepaired after foreseeing high repair costs or a time-consuming repair process. For example, one female participant stated that she generally gave up on getting items fixed if the repair took four days or more or would cost more than buying new products (unless the old items had a certain level of sentimental value).

At the same time as looking for information about a specific repair route, participants also considered alternative options, including other repair routes or replacing the product. Most participants identified repair costs as a factor when considering whether or not to choose commercial repair (SC2.7 and SC2.8). Some participants refrained from self-repair through a concern that disassembly would cause further damage or void the manufacturer’s warranty (SC2.8).

A growing realisation of the need for specialist tools (SC2.8), either during or after getting more information, could hinder repair activity at this stage. Sewing machines were usually required for DIY clothes mending and furniture reupholstery (though sewing machines for the former were said to be less expensive than for the latter, which may require industrial models). In some cases, specialist screwdrivers were required for self-repairing EEE.

Additionally, evaluation of different repair routes could be informed by the expected outcomes (SC2.7 and SC2.8). There seems to be *‘a right way or a couple of ways to fix electrical items’*. By contrast, clothing and furniture repair could deviate from the original design or intention and be associated with upcycling or changing products’ structure or appearance, depending on the owner’s taste and needs.

### Stage 3 – Repair in Action

**Fig. 7:**
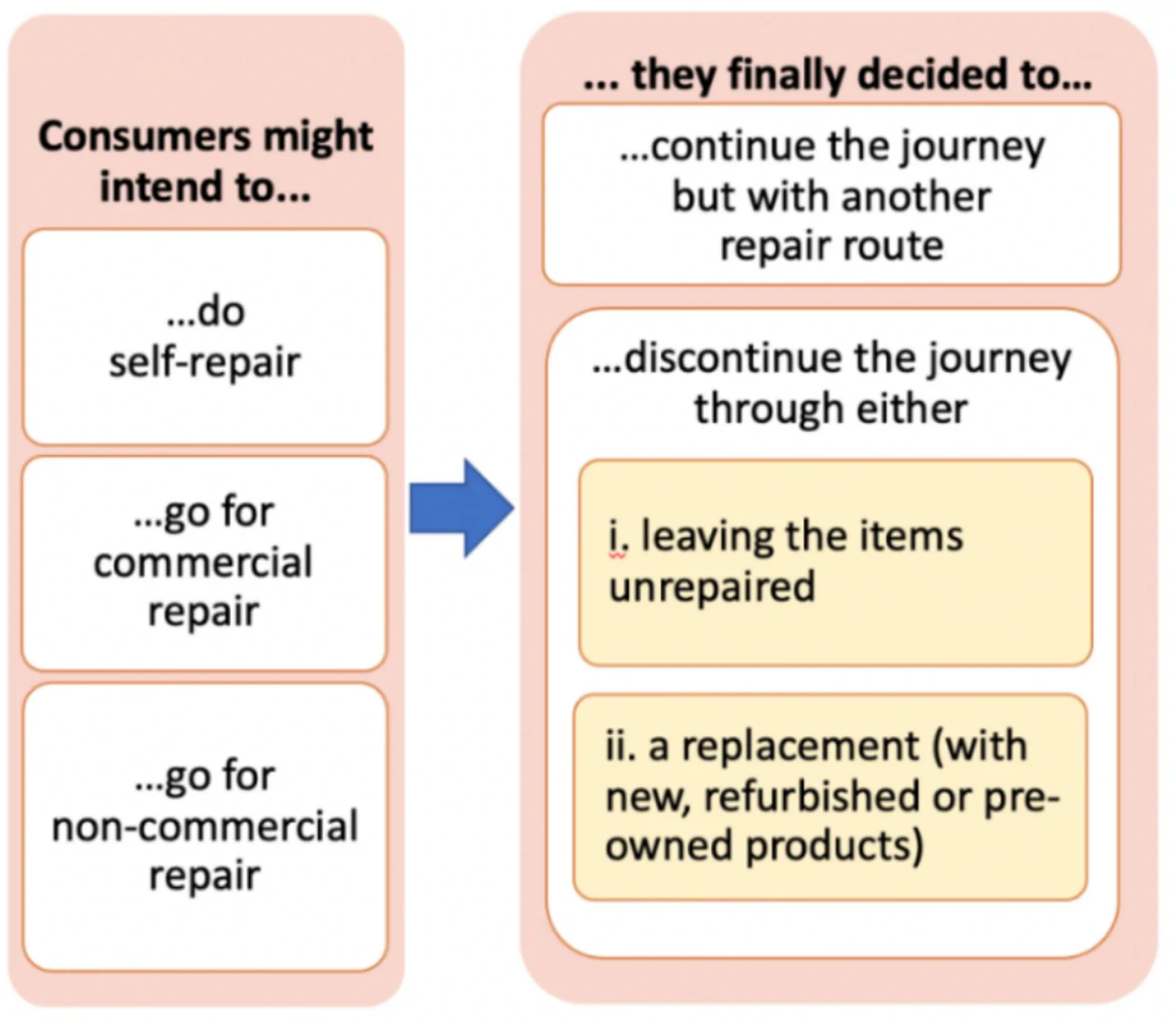
Intention-behaviour gaps at Stage 3

Consumers might intend to choose one of the three repair routes but later decide either to discontinue the journey or to proceed using another route. As shown in Fig. 7, intention (SC3.1 in Table 2) and behaviour (SC3.2) might align or diverge due to unforeseen challenges.

The data suggested that Stage 3 could occur alongside, after or without Stage 2. As Stage 2 helped them to make informed decisions, several participants were more confident in their chosen repair routes at Stage 3. However, others had to look for further information when starting or during the actual repair. There were similar obstacles to repair, including high cost and lack of time and skills (SC3.3), at Stage 3 compared to the previous stage. Over-estimation of personal skills or underestimation of repair time and cost (SC3.4) at a previous stage could influence consumers’ behaviour at Stage 3, including their decision to proceed or discontinue their repair journey.

The convenience of DIY repair was often influenced by the availability of tools and support from others (SC3.4). For instance, if there were no DIY stores near a person’s property or a female participant could not borrow tools from her son, their broken items would be left unrepaired or fixed by commercial repairers. Likewise, another female received both mental and physical support, such as getting encouragement, tools or parts from a flatmate – a skilful seamstress – and translating repair intention into action (SC3.4) for her worn clothes and damaged furniture. Similar support could be found from family members or at community repair events such as local repair cafes. A female participant often got help from her mother for mending clothes and from her granddad for repairing EEE. Male participants were less likely to mention difficulty accessing tools or requiring assistance.

Giving up the repair journey was sometimes associated with unexpected situations such as spare parts or tools being out of stock, accidental damage in the disassembly process, long queues for customer service, or fitting non-OEM components (SC3.4). The data indicated concerns regarding voiding warranties, safety issues, or causing additional damage when installing non-OEM components. As a result, opting for authorised repair services, particularly for EEE and furniture items, became more prevalent.

Some products could have been repaired by consumers if they had been designed for ease of repair and spare parts were available to the public: *‘Variability and complexity in parts are the biggest challenges to repair electronics… New items are designed and built to be cheap, not repairable. You can't get repair done without damage.’.* Some participants expected to have spare parts available for self-repair of electrical and electronic equipment (EEE) for at least five years. In contrast, for clothing items such as zippers, buttons, or patches, and for furniture components such as brackets and upholstery springs, the anticipated availability was one to two years from the product's introduction. In other words, designing products for easy repair and ensuring spare parts are accessible to the public could impact both the intention to repair and the conversion of that intention into actual behaviour (SC3.3 and SC3.4).

Growing up in families where repair was done inspired some participants to act similarly, and their intention to repair could remain at a high level throughout the repair journey (SC3.3).

In every focus group some participants doubted the competence of repairers in independent repair stores or retailers. In one case this concern had resulted in a replacement being preferred. DIY repair could sometimes be preferred because people had *‘a lot more access’* to online repair manuals or more easily sought help from people *‘who have enough time and enthusiasm to look at the problem’*, such as volunteers at repair cafes (SC3.4). Another female participant said that most of the retailers she visited had to send broken items away for repair, to either OEM or central service hubs, due to the lack of in-store facilities (SC3.4). This made her prefer local independent repair shops, where repair could be done promptly, sometimes within an hour.

### Stage 4 – Post-Repair Evaluation

**Fig. 8:**
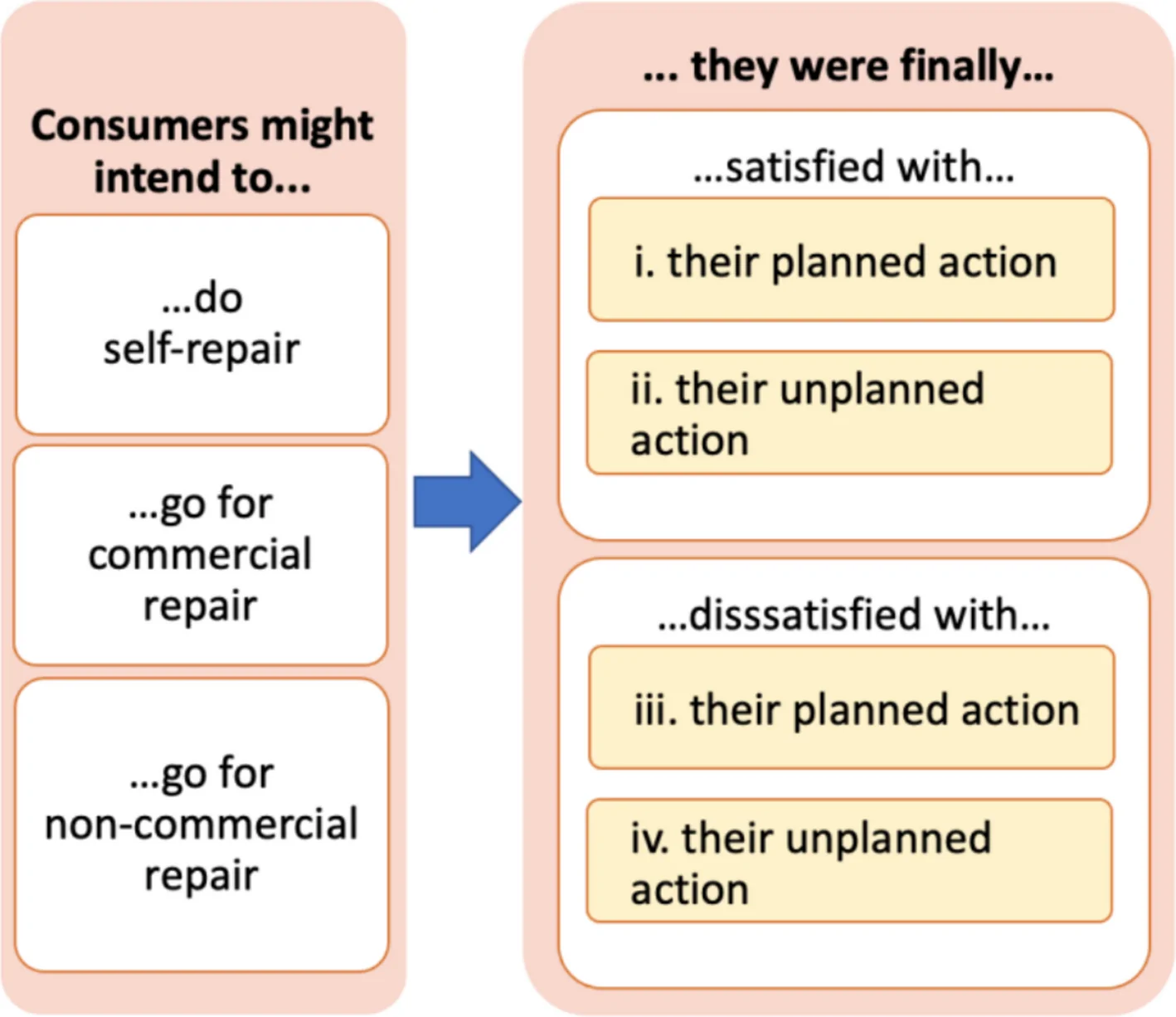
Intention and satisfaction gaps throughout the repair journey

During this stage, participants examined the complete journey to pinpoint distinctions in various aspects such as any changes in their decisions and at earlier stages. Discrepancies could also be found between initial expectations and satisfaction with the repair results, and between their initial intentions and the actions which they took that led to these outcomes. As shown in Fig. 8, intention (derived from SC4.1 in Table 2) and actual behaviour (SC4.2) are compared to evaluate satisfaction or dissatisfaction.

The rest of this section discusses factors influencing satisfaction or dissatisfaction with consumer repair decisions for different repair routes (coded SC4.3 and SC4.4 in Table 2). Satisfaction with self-repair decisions was reported to be generated from the enjoyment in undertaking self-repair, the cost saving over replacement and extending the product lifetime. For example, a female participant was grateful to her flatmate for loaning a sewing machine and sharing textile offcuts, which generated her interest in and a habit of self-repair rather than seeking local service providers. What mattered most to participants was whether the functionality of products returned after repair. Generation Y participants tended to associate their reasons for repair with environmental sustainability, whereas Generation X participants were primarily motivated by cost savings.

Most participants who succeeded in fixing a broken item indicated that they would be willing to self-repair the same or similar items in future. However, several regretted their decision and wondered if commercial repair would have been a better choice. The key reasons for this were a recurrence of faults or inability to resolve the problem. For instance, one female participant regretted attempting to shorten curtains, which she considered a waste of time and resulted from *‘overestimating [her] abilities’* and caused them further damage. She finally took them to a seamstress, which she did not regret.

Two participants criticised manufacturers for the high cost of spare parts for DIY repair, which made this repair route uneconomical and forced them to use authorised services. Others indicated that repetitious faults were caused by *‘low quality second-hand parts’* or that items had *‘nearly ended (their) lifetime’.* Moreover, a female participant indicated that satisfaction with repairing broken items could be achieved if the actual time did not exceed the time estimated based on the suggestions from online repair instructions, friends or family members (at Stage 2).

Most participants got items fixed by other people (e.g. commercial repairers or volunteers at repair cafés) only after realising they were not able to do self-repair. This sometimes resulted in unsatisfactory repair outcomes. Other participants had been frustrated when they had spent money on repair services or spare parts, but the quality of the repair did not meet their expectations. In some cases, the same faults recurred.

In addition to the challenges to self-repair such as knowledge, experience and confidence concerns of product owners, one male participant added that situational factors, such as the unavailability of specific parts, influenced his decisions for self-repair. He sometimes felt that he had no other choice than *‘sending items back to manufacturers’* for repair, and he then accepted any outcome thereafter.

It was evident in the group discussions that previous experience can influence decisions and actions undertaken in future repair journeys either positively or negatively, regardless of the repair route. For example, a few participants preferred self-repair due to losing trust in commercial services; whilst one participant admitted to not being willing to self-repair electronics again, judging it to be a waste of time.

Participants’ satisfaction with repair outcomes also varied across different product types. Repair of clothing and furniture products was *‘a bit more bespoke’*, which *‘makes them individual and improves some of the original… in a unique way’* compared to EEE. For this reason, a male participant was *‘more than happy’* with his repair work, especially with chairs in his pub that he repaired to *‘a standard better [more steady] than the original.’* In contrast, a female participant was not really satisfied with the aesthetic of her sofa cushion after self-repair but was grateful that it at least extended the life of the item without her paying for a service. Two other male participants shared a similar mindset. For example, *‘I don't think I've been dissatisfied but, at the same time, I have that thing of knowing that it would look better if a professional had done it’.* Another male participant believed that the replacement of various components in EEE could restore the functionality of entire items and, for that reason, did not regret any repair journey that he had experienced.

## Discussion

**Fig. 9:**
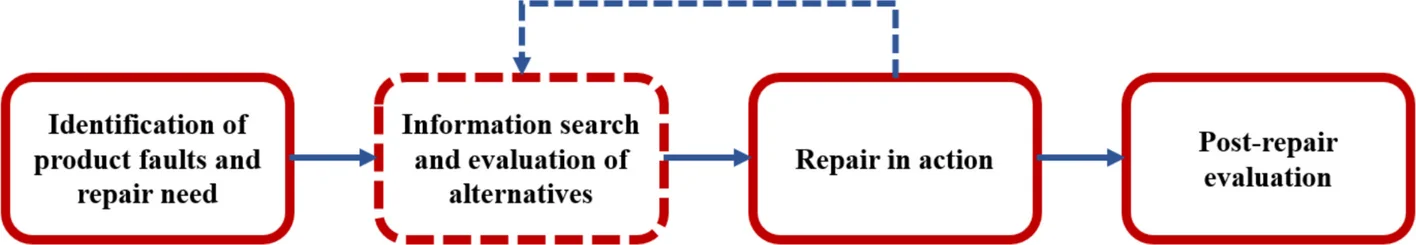
Finalised consumer repair journey

The findings from this research explain what could affect consumer repair journeys. Figure 9 presents the finalised consumer repair journey, comprising four key stages. Stage 2 sometimes could be skipped if consumers have already previously encountered the same or similar repair experience. Moreover, consumers could enter into in Stage 3 ‘Repair in action’ but later return to Stage 2 to seek further information, either to continue with the repair process or explore other repair routes.

The first sub-section below discusses the initial two themes that emerged from the findings, which address similarities and differences in factors that influence intention during the first three stages (i.e. before and during the repair action), and how intention translates into behaviour during them. The second sub-section discusses the third theme, which addresses factors that could influence consumer satisfaction regarding the translation of intention into behaviour when considering different repair options.

### Factors Influencing Intention and the Translation of Intention into Behaviour

This study developed and introduced a new conceptual framework, the consumer repair journey, to better understand consumer repair intention and behaviour. Table 4 provides a summary of the factors (e.g. what could influence intention or the translation of intention into behaviour) associated with the first two themes which emerged from the analysis. These factors can influence the consumer repair journey in different ways at various stages, particularly during the first three stages – both before and during the repair action. As the study examines repair intention versus actual repair behaviour, this approach explicitly links with Theory of Planned Behaviour [2]. Therefore, the factors in Table 4 are mapped to the components of this theory: attitudes, subjective norms and perceived behavioural control to behavioural intention, which then influences action. Particularly, attitude (A) could be a positive or negative evaluation of undertaking the repair. Subjective Norms (SN) could be perceived as social pressure or influence to repair or not to repair), and Perceived Behavioural Control (PBC) could be perceived as ease or difficulty in performing the repair, linked to skills, resources, and constraints. Table 4 also includes a set of Contextual Factors (CF), which account for external conditions that can facilitate or hinder behaviour.

**Table 4:** Factors influencing intention and the translation of intention into behaviour before and during repair action the consumer repair journey

	Theme 1: Factors influencing initial intention during the consumer repair journey	Theme 2: Factors influencing the translation of intention into behaviour during the consumer repair journey
Stage 1: Identification of product fault and repair need	• Levels of competence in repair (PBC) • Confidence about successful outcomes (PBC) • Interests in repair (A) • Nature of product faults/ product design complexity (CF)	
	• Personal preference for replacement as cheaper and simpler (PBC)	• Fear of further functional, or aesthetic, damage (A) • Time required for identifying product faults (PBC) • Special tools required for identifying product faults (CF)
Stage 2: Information search and evaluation of alternatives	• Interests in repair (A) • Repair costs (A) • Reliance on friends’ and family members’ expertise (SN) • Expected repair outcomes (A)	
	• Doubts about the usefulness of manufacturer manuals (A)	• Concerns about safety issues or voiding manufacturer’s warranty (A) • Variability in time and effort required for information search (PBC) • Challenges in finding reliable repair service providers (CF)
Stage 3: Repair in action	• Product design complexity	
	• Repair costs (A) • Time and skills required for repair (PBC)	• Over-estimation of personal skills (PBC) • Underestimation of repair time and cost (PBC) • Variability in time and effort required for actual repair work (PBC) • Doubts about repairers’ competence (A) • Unavailability of spare parts or tools (CF) • Limited in-store repair facilities (CF) • Unexpected situations for in-store professional repair (e.g. Limited facilities, long queues) (CF)

Upon examining the factors within the consumer repair journey (Table 4), several interconnected issues emerge, significantly influencing consumer intention and the translation of intention into behaviour. Initially, levels of competence in repair, confidence about repair outcomes and a lack of interest in repair (Stage 1) might lead to reluctance in investing time and effort to identify faults and pursue repair options. Significantly, males were more likely to identify and engage with faults in EEE, while females showed more interest and competence in repairing clothing, with no notable gender differences observed for furniture. These findings align with a discovery made in previous research [26, 27, 46, 50, 54] indicating that women exhibit greater involvement in clothing self-repair practices. This is also reflected in the work of Fisher et al. [24], who showed that gender plays a critical role in shaping sustainable clothing consumption, including repair habits. [54] similarly noted that women face structural and psychological barriers to EEE repair, which limit their participation. The current study also revealed that disinterest often persists through the information search and evaluation of alternatives (Stage 2), as consumers may choose convenience or replacement over actively seeking repair solutions. Therefore, educational campaigns and repair workshops could be gender-sensitive, offering EEE repair skills to women and clothing repair resources to men to close competence gaps and foster greater repair interests.

The exploration of factors influencing intention and the translation of intention into behaviour highlights both shared and distinct impact factors between the two themes (Table 4). This study emphasises these differences, contributing to a deeper understanding of consumer intention and behaviour at various stages of the repair journey. For example, the formation of intention at the start of the repair journey (Stage 1) could be influenced by personal preference for replacement, which could be viewed as a cheaper and simpler option. Consumers might also abandon their initial intention to identify product faults themselves due to concerns about the time needed for diagnosis, the requirement for specialised tools, or the risk of causing further functional or aesthetic damage. For instance, fear of additional damage (Stage 1) could impact consumer confidence when repairing clothes or furniture, while data loss is a concern in the repair of EEE products. The complexity of product faults and design, especially in EEE, could heighten these concerns, particularly at Stages 1 and 3. This could lead consumers to underestimate the time and effort needed or overestimate their own skills, ultimately hindering the ability to translate repair intentions into actions at Stage 3. Furthermore, unlike the findings of previous studies on EEE repair [54, 60, 73], the current research indicates that age does not significantly influence the likelihood of engaging in repair activities, whether through DIY or professional services, across the three product sectors.

The data collected indicated that cost savings (among male participants) and waste reduction (more commonly emphasised by female participants) were primary motivations for repair. A previous study [56]found that women participants placed a higher value on sustainable behaviour such as recycling, reuse and repair. In the current study particularly, concerns about the costs of different repair routes at Stage 2 and Stage 3 could play a significant role in shaping consumer intention between repair and replacement. These gendered preferences suggest the potential value of tailored, marketing strategies, such as highlighting financial savings to appeal to men and emphasising environmental sustainability to resonate more with women.

For all three product sectors, the variability in time and effort required for information search or repair work (Stages 2 and 3) could be compounded by limited access to reliable repair information and services. Male participants tended to prefer online information sources, valuing the sense of challenge and independence that self-directed learning offered. They often expressed greater confidence in their existing knowledge and actively looked for tutorials and guidance through digital platforms. In contrast, female participants showed a stronger reliance on personal advice from friends and family members. This dependence on social networks during Stage 2 highlights the significant role of peer influence in shaping repair intention and behaviour, underscoring the importance of social support through the consumer repair journey.

As a result of limited access to reliable repair information and services, consumers at Stage 3, might realise they initially underestimated the costs and time involved or overestimated their personal repair skills. This misjudgement could be compounded by the variability in the time, cost, and effort required for the actual repair. These factors could also be influenced by the unavailability of spare parts or tools (regardless of the repair method) or by limited in-store repair facilities and unexpected challenges, such as long queues or insufficient equipment, when opting for commercial repair services.

Furthermore, consumer concerns across different stages of the repair journey reveal significant barriers rooted in trust, confidence and resource accessibility. At Stage 2, worry about voiding warranties, safety warnings, and the expense of parts and tools, particularly for EEE and furniture products, often inhibited consumers from translating repair intention into actual behaviour. Trust in the assistance of manufacturer manuals might also deter consumers from pursuing self-repair at Stage 2. In the context of commercial repair, earlier studies have shown that the relatively low replacement and high repair costs significantly influenced the decline in demand for repair services [47]. This study investigates further into additional factors, focusing on how consumers make decisions to repair or replace. For instance, difficulties in finding reliable service providers could prevent consumers from following through with the repair at Stage 2.

Similar to the findings of previous studies [15, 65], doubts about repairers’ competence further compound trust issues, discouraging engagement with commercial repair services, particularly at Stage 3. Additionally, the unavailability of spare parts or appropriate tools emerges as a major obstacle, underscoring the importance of resource accessibility and technical support in enabling successful repair outcomes. As male participants appeared to have better access to tools, whilst more female participants demonstrated greater dependence on others for tool use, expanding tool-sharing initiatives or rental schemes could help address this gap. Developing inclusive community repair infrastructure could further support participation in repair activities across genders.

In general, addressing the key patterns between different stages in the consumer repair journey necessitates a holistic approach that considers the interconnected nature of challenges and the diverse factors influencing consumer repair intention and the translation of intention into behaviour. Table 5 summarises key patterns in the findings by gender and suggests potential intervention implications tailored to different genders.

**Table 5:** Key patterns and implications by genders

Area	Male-dominant patterns	Female-dominant patterns	Intervention implications
Repair interest in product type	EEE	Clothing	Offer skill-specific training and cross-gender repair promotion
Motivations	Financial savings	Environmental values	Tailor marketing such as cost for men and sustainability for women
Information source preferences	Online (sense of challenge and independence)	Personal advice (friends and family)	Combine digital and in-person support
Tool access	Better tool access	Tool dependence on others	Expand tool sharing or rentals, build inclusive community repair infrastructure

### Factors Influencing the Consumer Satisfaction with the Translation of Intention into Behaviour

The findings from this study suggest that past experiences with repair services could impact future decisions, emphasising the need to address challenges encountered during the consumer repair journey. While past literature has explored opportunities and challenges in product repair [5, 19, 26, 27, 47, 49, 69, 73], it has not fully examined what influences consumer satisfaction with their repair decisions.

**Fig. 10:**
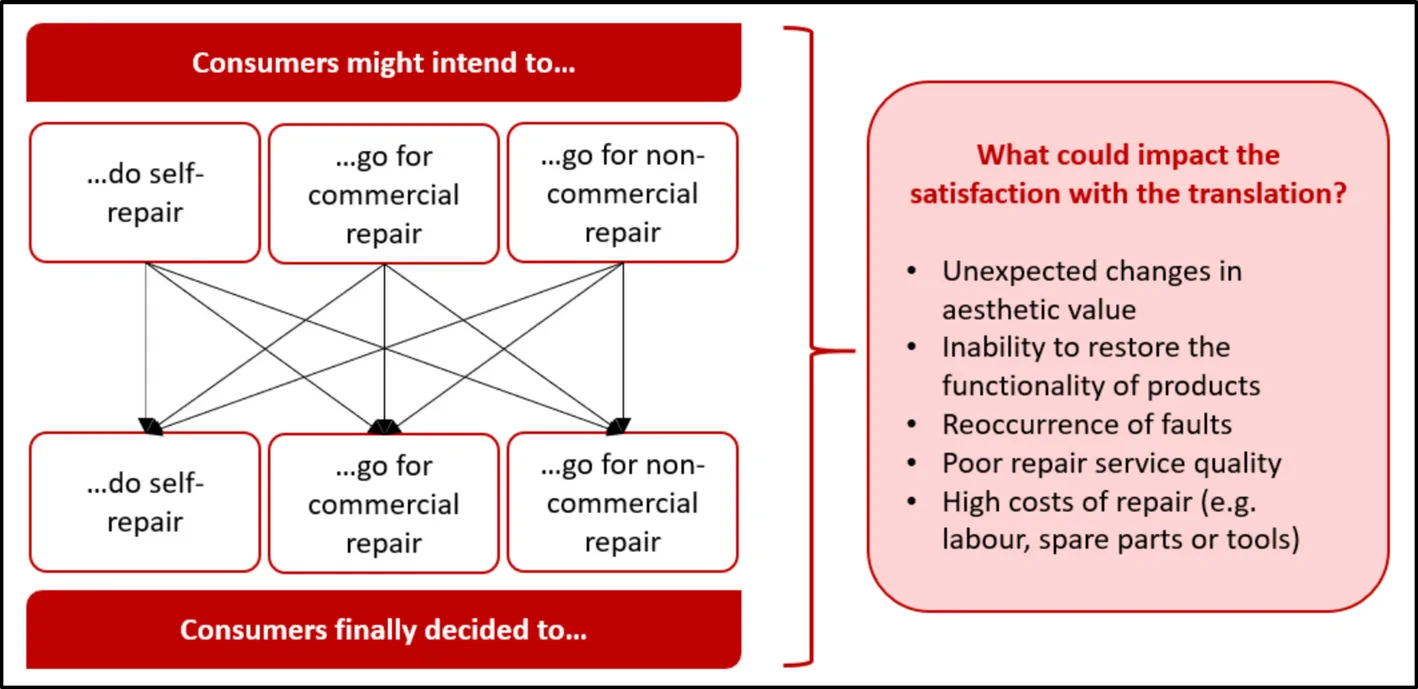
Factors influencing the satisfaction with the translation of intention into behaviour

To address this gap, Fig. 10 summarises the various ways in which different intentions could lead to different behaviours, depending on the chosen repair routes (e.g. self-repair, commercial repair, or non-commercial repair). The study indicates that the intention to choose a particular repair route might either remain consistent or shift to another option. Factors influencing satisfaction with these transitions were identified during group discussions and are also represented in Fig. 10 (the right-hand box).

Post-repair evaluation (Stage 4) depends on whether the repair successfully restores both functional and aesthetic value. While many consumers may be satisfied if EEE products function as they did before, those repairing clothing and furniture often seek both functional and aesthetic restoration. Additionally, perceptions of value restoration at Stage 4 could be shaped by factors such as restored functionality, changes in aesthetic appeal, and the recurrence of faults. Concerns about repair costs, which heavily influence the decision between repair and replacement in earlier stages, continue to have a significant impact on consumer satisfaction with their choice at this stage.

Understanding these key factors that shape consumer satisfaction is essential for improving their overall repair experience throughout the repair journey. For example, by addressing concerns about the cost of repairs, offering clearer guidance on repairability, and ensuring the availability of spare parts and reliable services, businesses and repair providers would foster greater confidence in consumers' decisions to repair rather than replace.

Additionally, providing transparency in pricing and repair processes, or enhancing post-repair support, might further improve customer satisfaction. A holistic approach by businesses to consumer repair experiences could also encourage more sustainable behaviour, making the decision to repair feel less daunting and more rewarding, especially when consumers feel their investment of time, effort, and money has restored both the functionality and value of the product.

### Recommendations for Business Interventions

Consumers could have varying intentions, either following through with their initial plan or discontinuing the repair journey at some point (as shown in Fig. 5–8), which are influenced by a range of factors. These impact factors, along with those affecting customer satisfaction, differ based on the specific stage of the repair journey (as detailed in Table 4 and Fig. 10). Appropriate and sufficient interventions and support from businesses are crucial if consumers are to make informed repair decisions. Based on the key impact factors found at each stage of the consumer repair journey and with a consideration of different product sectors, two groups of recommendations are proposed for business interventions: these relate to (1) the availability of and access to repairable products, and (2) repair services and customer support.

#### Recommendation 1

The importance of enhancing the availability and accessibility of repairable products has been underscored. *Manufacturers, brands, and retailers should consider design strategies such as disassembly, standardisation of parts, simplification, and modularity to facilitate repair, and promote these product features.* This recommendation aligns with initiatives outlined in a consumer behavioural study addressing product durability and repairability [21] and a study conducted by the Reuse and Recycling EU Social Enterprises network [68]. For example, Fairphone, one of the few sustainable mobile phone manufacturers, produces modular smartphones specifically designed for repair and longevity [32]. The current study further highlights that product design plays a significant role in translating intention into behaviour during the consumer journey. In particular, design complexity, as indicated in Stage 1 and Stage 3 of the repair journey (Table 4), could influence the consumer's ability to diagnose product faults and influences their willingness to repair, especially when time or special tools are required to identify the issue. ​Therefore, manufacturers should prioritise designing products with modularity and ease of repair in mind; this approach could empower customers to confidently diagnose issues and attempt repairs. As shown in Table 5, differences in tool access and repair interest between genders suggest that simplifying product disassembly and fault diagnostics could help bridge these gaps. Collaborating with design experts and conducting usability testing could make repair more accessible for both males and females, enabling customers to carry out repair independently and improving their overall experience and satisfaction.

The present study suggests that *businesses should initiate marketing campaigns emphasising the benefits of repair, such as cost savings, reduced environmental impact, and extended product lifespan, all of which influence repair intentions.* This study added to findings of previous research [26, 27, 40]. In particularly, showcasing repairable product value could engage consumers, addressing the lack of repair interest from Stage 1 and encouraging repair consideration. The current research contributes to the existing knowledge by indicating that emphasising EEE product functionality and functional and aesthetic value for clothing and furniture positively impacts repair intention, the translation of intention into behaviour and overall satisfaction. Additionally, promoting online repair communities fosters the exchange of knowledge and peer influence, particularly among Generation Y, who are driven by their environmental sustainability objectives. This potentially signifies a systemic implication for improving resource utilisation and extending product lifespan in consumption practices, given this generation's inclination towards online platforms and repair for environmental sustainability purposes, as highlighted in the current study. In practice, Patagonia [59], an American outdoor clothing brand, exemplifies this approach by offering free repairs and DIY repair kits to promote repair as a sustainable act and reinforce its brand values.

Furthermore, Stage 2 highlights the challenges consumers face when searching for repair information. Overwhelming or unclear manuals, along with concerns about safety or voiding warranties, often prevent consumers from committing to repairs (Table 4). *Easy-to-follow repair guides, suited for both DIY enthusiasts and commercial repair,* could ease these concerns, maintaining consumer commitment to repair*.* Recognising the lasting impact of training, as highlighted in a previous study [40], businesses should prioritise raising awareness of product repairability and its benefits*. Offering workshops and tutorials during Stages 1 and 3* could enhance consumer skills. These sessions should address specific barriers encountered at each stage (Table 4) and cater to diverse gender interests and abilities in repairing different product types. Improving tool access for females in EEE repair and encouraging great engagement of males in clothing repair (Table 5).

Lastly, the availability of spare parts is another significant barrier, as noted in Stage 3 (Table 4). Consumers often face shortages, limited in-store facilities, and unexpected situations that disrupt repairs. Manufacturers should address this by improving spare part access, ensuring availability at purchase and during product use. *Implementing standardised parts across product lines and partnering with third-party repair services* could streamline the procurement process for consumers, reducing barriers to repair and positively influencing consumer intention and the translation of intention into behaviour. A study in Iran [38] highlights that modular and standardised design in electronics enables continuous repair and maintenance, significantly enhancing product longevity. The EU’s recent eco-design regulations (European [22]) apply to a limited range of products, specifically smartphones, tablets and cordless phones – requiring key spare parts to be available for at least 7 years. As proposed during the group discussions, consumers expected spare parts to be accessible for self-repair of other EEE products for at least 5 years. In contrast, for clothing items (such as zippers, patches, and buttons) and furniture (including screws, brackets, and upholstery springs), the anticipated timeframe could be 2 years from the introduction of the models. IKEA [37] exemplifies this approach by offering free spare parts such as screws, bolts and dowels, along with a range of larger spare parts. The company also promotes DIY repairs via manuals and instructional videos.

#### Recommendation 2

Taking into account the challenges faced at each stage of the consumer repair journey and the factors influencing consumer satisfaction (Fig. 10), the current research suggests that *manufacturers, brands, and retailers should address several key areas to enhance access to and quality of repair services and customer support*. Firstly, they should *offer responsive online services, improve staff expertise, and expedite repair service turnaround times*. These efforts are crucial in shaping consumers' perceptions of repair services, which should ideally be straightforward, prompt, and effective. For instance, Stage 3 highlights issues such as long repair times, uncertainty about repairer competence, and the unavailability of spare parts, which could deter consumers from completing repair (Table 4). The research also highlights how past experiences with repair services can influence future decisions. If a customer faces difficulties, such as limited in-store repair facilities or unexpected repair challenges, during a previous repair attempt, they may be less inclined to choose repair over replacement in the future​. Therefore, businesses must resolve these problems early in the consumer journey to build trust and encourage repeat repair.

The study aligns with previous research [44, 72] indicating that online resources positively impact the purchasing process and encourage sustainable behaviour. Stage 2 in Table 4 reveals that the variability in time and effort needed to search for reliable repair service providers and concerns over safety or voiding warranties often frustrate consumers. Both Generations X and Y in the current study show a preference for accessing repair instructions and recommendations for reliable commercial services online. Issues such as the unavailability of local repair services, long queues for customer support, and urgent product needs often deter individuals from seeking commercial repair, as outlined in Stage 3 (Table 4), potentially leading to a habit of avoiding commercial repair altogether. By providing *responsive online support, competent staff, and swift product turnaround times,* businesses would significantly enhance the convenience of commercial repair.

Businesses should also *adopt transparent pricing models and offer upfront estimates for repair services* to counteract perceptions of high repair costs (particularly at Stages 2 and 4). *Flexible payment options and loyalty programs* could further mitigate concerns about affordability and encourage consumers to choose repair over replacement.

Furthermore, this study validates conclusions drawn from prior research while broadening the scope to include product sectors beyond those addressed previously, such as smartphones and washing machines [26, 27]. It emphasises that repair services should prioritise transparency, convenience, and reliability, demonstrated through certifications, customer testimonials, and adherence to safety protocols. Figure 10 highlights how ensuring quality assurance measures and offering warranties or satisfaction guarantees could boost consumer confidence. *Providing avenues for consumers to provide feedback and report any post-repair issues* could help identify areas for improvement and enhance overall customer satisfaction, thereby streamlining the post-repair evaluation process (Stage 4).

## Conclusion and Further Research

This study has provided important exploratory, insights into the consumer repair journey and potential business opportunities to promote product repairability, considering the various stages of the journey, different repair routes, and multiple product sectors. It highlights the significance of product design, the availability of repair information and spare parts, and the quality of after-sales services in shaping intentions to repair and influencing consumer behaviour at each stage of repair journeys.

Its findings offer guidance for industry professionals on implementing interventions that go beyond product design. Such interventions should include comprehensive support services that accompany a product throughout its lifecycle, ultimately facilitating the repair experience and improving consumer satisfaction. The insights are thus potentially useful for businesses looking to strengthen consumer follow-through from repair intention to action. By incorporating the insights into their practices, businesses could align with the growing global movement towards the ‘right to repair’, as advocated by the European Commission [22] and more recently by Right to Repair Europe [63].

Strategic interventions such as introducing innovation in product design, communication strategies, and instructional workshops targeted at improving product repairability (Recommendation 1) may strengthen brand reputation and amplify customer preferences for both brands and their products. Enhancing access to spare parts (Recommendation 1) and improving repair services and customer support (Recommendation 2) could boost customer satisfaction with post-purchase services. These measures have the potential to create supplementary revenue streams, driving growth in sustainable, repair-focused enterprises across various product sectors.

Given the findings, and noting the constraints of the research, it is evident that further studies are required in several areas. Firstly, quantitative inquiries into consumer repair journeys at either the national or EU level should be conducted, considering demographic factors such as generations beyond Generations X and Y, as well as varying income levels and cultural contexts that might influence repair behaviours. Secondly, an exploration of the applicability of the 'consumer repair journey' concept to product repairability and repair services across more product sectors is warranted. Experiments involving product prototypes designed with repairability strategies are essential, accompanied by investigations into consumer repair journeys associated with these products. Finally, there is a necessity to explore the business management implications and advantages for various stakeholders in the adoption and implementation of business innovations aimed at enhancing product repairability and repair services.
